# Altered Food Habits? Understanding the Feeding Preference of Free-Ranging Gray Langurs Within an Urban Settlement

**DOI:** 10.3389/fpsyg.2021.649027

**Published:** 2021-04-26

**Authors:** Dishari Dasgupta, Arnab Banerjee, Rikita Karar, Debolina Banerjee, Shohini Mitra, Purnendu Sardar, Srijita Karmakar, Aparajita Bhattacharya, Swastika Ghosh, Pritha Bhattacharjee, Manabi Paul

**Affiliations:** ^1^Department of Environmental Science, University of Calcutta, Kolkata, India; ^2^Centre for Mathematical Biology and Ecology, Department of Mathematics, Jadavpur University, Kolkata, India; ^3^Systems Ecology and Ecological Modelling Laboratory, Department of Zoology, Visva-Bharati University, Santiniketan, India; ^4^Department of Environmental Science and Engineering, Indian Institute of Technology (Indian School of Mines), Dhanbad, India; ^5^Department of Biological Sciences, Indian Institute of Science Education and Research Kolkata, Nadia, India

**Keywords:** gray langur, free-ranging, urbanization, co-existence, feeding preference

## Abstract

Urbanization affects concurrent human-animal interactions as a result of altered resource availability and land use pattern, which leads to considerable ecological consequences. While some animals have lost their habitat due to urban encroachment, few of them managed to survive within the urban ecosystem by altering their natural behavioral patterns. The feeding repertoire of folivorous colobines, such as gray langur, largely consists of plant parts. However, these free-ranging langurs tend to be attuned to the processed high-calorie food sources to attain maximum benefits within the concrete jungle having insignificant greenery. Therefore, besides understanding their population dynamics, the effective management of these urbanized, free-ranging, non-human primate populations also depends on their altered feeding habits. Here, we have used a field-based experimental setup that allows gray langurs to choose between processed and unprocessed food options, being independent of any inter-specific conflicts over resources due to food scarcity. The multinomial logit model reveals the choice-based decision-making of these free-ranging gray langurs in an urban settlement of West Bengal, India, where they have not only learned to recognize the human-provisioned processed food items as an alternative food source but also shown a keen interest in it. However, such a mismatch between the generalized feeding behavior of folivorous colobines and their specialized gut physiology reminds us of Liem's paradox and demands considerable scientific attention. While urbanization imposes tremendous survival challenges to these animals, it also opens up for various alternative options for surviving in close proximity to humans which is reflected in this study, and could guide us for the establishment of a sustainable urban ecosystem in the future.

## Introduction

The global urban human population is set to reach the 5 billion mark by 2028 (ONU, 2018), facilitating urban sprawling and subsequently contributing to natural habitat loss worldwide at an unprecedented rate. This is expected to affect a large number of animals whose habitat ranges overlap with urban areas (Mcdonald et al., 2008; He et al., 2014; Martinuzzi et al., 2015; Murray and St. Clair, 2015). Habitat fragmentation and encroachment due to such urban expansion, which is often irreversible, has forced many homeless animals to live in close proximity to humans (Bateman and Fleming, 2012), giving rise to frequent human-animal conflict (Messmer, 2000; Omondi, 2004; Woodroffe et al., 2005; Devi and Saikia, 2008). At the same time, some of these animals have also shown considerable behavioral adaptations like altered nesting or denning habits, vocalization, migratory activities, mating and breeding patterns, feeding behavior (Kettlewell, 1961; Able and Belthoff, 1998; Estes and Mannan, 2003; Slabbekoorn and Peet, 2003; Swedell et al., 2011; Lowry et al., 2013) together with life history modification to survive amidst anthropogenic stress. Such anthropogenic stress often creates unpredictable selection pressure on these urban animals, leading to a sharp decline in species richness and composition within an urban ecosystem (Vitousek et al., 1997; Kumara and Singh, 2004; Singh and Raghunatha Rao, 2004; Fuentes, 2012; Kale et al., 2012; Paul et al., 2016; Erinjery et al., 2017). However, despite significant loss of biodiversity, urban expansion offers various high-calorie resource options to the generalist species who have higher dietary as well as foraging plasticity, and therefore, can adjust more readily to the altered habitat in contrast to the specialists (Vázquez and Simberloff, 2002; Fisher and Owens, 2004). Moreover, such anthropogenic food sources remain available throughout the year, thus providing a risky yet reliable and easily accessible resource option which is thought to be one of the major driving forces behind human-animal co-existence within urban settlements (Bateman and Fleming, 2012; Lowry et al., 2013; Widdows et al., 2015; Thabethe and Downs, 2018). In some cases, urban-dwelling free-ranging animals have been shown to acquire a preference toward anthropogenic food items to minimize their foraging activities, so that could invest more energy and time in nurturing social relationships which is essential to attain better fitness benefits (Saj et al., 1999; Hoffman and O'Riain, 2012; Sha and Hanya, 2013; Bryson-Morrison et al., 2016, 2017; Thatcher et al., 2019).

India has more than 400 mammalian species, of which 17 are non-human primates with different conservation status (Molur et al., 2003; Karanth et al., 2010; Kumara et al., 2010) who have ecological as well as socio-cultural importance. Three of these non-human primate species i.e., Rhesus macaques (*Macaca mulatta*), bonnet macaques (*Macaca radiata*) and gray langurs (*Semnopithecus entellus*) are frequently found in Indian cities, market places, temples, roadside settlements, etc., where they are often provisioned with human offered food items and space, worshiped and protected by *Hindus* (Sharma et al., 2011). Their wide distribution range and various feeding habits reflect their generalistic nature where they use a handful of novel strategies such as “coo-calls,” begging gestures, car raiding, etc. to acquire the available food items directly from humans (Sinha, 2005; Arbib et al., 2008; Sha et al., 2009; Deshpande et al., 2018). However, such close human-animal interaction is often lethal, affecting their chances of survival within urban ecosystems (Grinder and Krausman, 2001; Vijayan and Pati, 2002; Gosselink et al., 2007; Paul et al., 2016). Furthermore, these high-calorie processed food items could have a substantial effect on the physiology of these animals underlying their behavioral patterns, thereby reshaping intra and inter-specific group dynamics in contrast to their natural counterparts (Orams, 2002; Higginbottom and Scott, 2008; Trave et al., 2017). In this scenario, it is imperative to understand how the oppression of urban expansion has thinned down the natural resources and influenced the lives of these animals, leading to urban-adaptation in these species.

While several studies have been carried out on the naturally omnivorous macaques (Oppenheimer, 1977; Goldstein and Richard, 1989; Laska, 2001; Ganguly and Chauhan, 2018), to understand their opportunistic feeding behavior to co-exist with human settlements, there has been no study yet to quantify and compare the dietary preference of folivorous gray langurs in urban areas. Due to their deity value, this species is endowed with ample human provisioning. However, their specialized tripartite stomach structure largely aids in the digestion of a leafy diet (Bauchop and Martucci, 1968; Caton, 1999). Moreover, unlike the terrestrial macaques, the arboreal nature of gray langurs (Khanal et al., 2018) is also barring them from availing enough human provisioning which could supplement their cellulose-based diet. Therefore, it seems to be all the more difficult for these large-bodied colobines to obtain sufficient resources that could be invested in maintaining reproductive fitness within an ecosystem where their natural food options are either unavailable or scarce to support their energy demand.

Gray langurs (*Semnopithecus entellus*), commonly called Hanuman langurs have colonized various parts of the Indian subcontinent, ranging from the desert to forest fringes, and have lived with a diversified resource structure and human interference (Oppenheimer, 1977; Ashalakshmi et al., 2014; Chetan et al., 2014). In comparison to the other species of langur, social organization of the gray langur is highly flexible (Newton, 1988; Caton, 1999; Sterck, 1999; Rajpurohit et al., 2006) and is often modified by the male-male competition followed by infanticides (Hrdy, 1974; Broom et al., 2004; Sharma et al., 2010). Besides unimale-bisexual troops, all-male bands are also common in these gray langurs (Rajpurohit et al., 2003). Even though they exploit a wide range of plant species including various plant parts, only a fraction of these so-called “Least Concern species” (IUCN 2003) can reach their reproductive age, which is again expected to reduce due to the adverse effect of urban encroachment (Kumara et al., 2020). On the contrary, such urban settlements provide easy access to various anthropogenic low-fiber food sources which are mostly processed yet offer high-calorie to these ruminant folivores (Sayers, 2013). Few articles have reported human-langur cooperation through the food provisioning in Indian cities and towns, portraying them as ecological generalists in terms of habitat and diet. This mismatch in their expected and observed diet has made them one of the prime examples of Liem's paradox which refers to the odd pairing of specialized anatomical features with a generalistic diet (Liem, 1980; Binning et al., 2009). However, it was later argued that such “asymmetry allows optimally foraging consumers to evolve phenotypic specializations on non-preferred resources without compromising their ability to use preferred resources” (Robinson and Wilson, 1998). Hence, the development of alternate feeding patterns in these urbanized free-ranging gray langurs demands considerable scientific attention which could provide important insights into their eco-ethological adaptation for better management and policy-making to develop a sustainable urban ecosystem. Several studies have manifested “food-resources” as one of the major contributing factors that limit group size and composition of primates (Chapman, 1990). The Van Schaik model posits that in folivorous non-human primates, the intra-group scramble feeding-competition leads to differential reproductive success which has an immense role in establishing the hierarchical relationship within the group (Van Schaik, 1989; Borries, 1993). Therefore, the feeding behavior of the gray langurs can provide interesting insights into the social dynamics, as well as the urban adaptation of the species. In this article, we have focused on the feeding preference of group-living, free-ranging gray langurs in the urban areas of West Bengal, India, which unraveled their behavioral plasticity to utilize anthropogenic habitats.

## Methods

### Study Area and Study Animals

Free-ranging gray langur groups were identified through regular census between September 2018 and December 2018 in various parts of West Bengal, India, of which three distinct langur groups [one in Dakshineswar (22.6573°N, 88.3624°E), one in Nangi (22.4973°N, 88.2214°E), and one in Sarenga, Nalpur (22.5307°N, 88.1840°E)] were selected for long-term observations, considering the various level of human interferences received by the gray langurs (Supplementary Data D1, D2).

### *Ad libitum* Study

Observers visited the areas at random times during the day and walked on all roads, by-lanes, and fields covered with vegetation of the above three selected urban settlements. Whenever a group of gray langurs was sighted, it was observed by using *ad libitum* for a minimum of 15 min to a maximum of 1 h, during which the observer recorded its location, group size, and behaviors (later categorized as either solitary or paired interactions; paired interactions were further subdivided into intra- and interspecific interactions). These behavioral interactions were used to prepare an ethogram which was used for long-term observations. We used physical and behavioral characteristics such as fur color, body size, locomotion, feeding, and sexual behavior to discriminate various life stages (Infant-dark fur color and fully dependent on adults for their movement and feeding; Juvenile-light fur color similar to adults but smaller in size and partially dependent on adults for their movement and feeding; sub adult- fully independent of adults but yet to attain sexual maturity, body size is typically in between that of juveniles and adults; adult-fully grown, independent individual who is sexually mature) of free-ranging gray langurs. Their ranges were accurately recorded during observation by using GPS- eTrex30, Garmin.

### Food-Census

We recorded the food habits of these three gray langur groups between September 2018 and December 2018 through regular census. Whenever any feeding behavior was observed, the observer recorded the details of food types (food-census), and source of the food item (whether foraged from roadside tree patches, raided or stolen from the agricultural field, household garden, or vegetable market, being provisioned by humans, etc.). We categorized these feeding behaviors into “solitary” (when a gray langur independently fed on some food item) and “interactive” (which involved interactions with other group members or humans). For interactive feeding behaviors, we recorded the identity of the “initiator” and “recipient.” “Initiator” represents the individual (gray langur or human) who initiates a behavior (stealing or provisioning) that facilitates gray langurs to acquire a food item, whereas “recipient” is the individual who received the behavior (human and gray langur are recipients for food stealing and food provisioning behaviors, respectively). The feeding repertoire of these gray langurs consisted of either unprocessed (various plant parts such as raw vegetables, fruits, tree leaves, flowers, etc.) or processed food items (factory-made such as bread, biscuit, peanut, chips, puffed rice, cake, etc.), which are associated with human presence and involve some degree of human interference within an urban ecosystem (Supplementary Data D3a,b).

### Food-Choice Test

**Figure 1:**
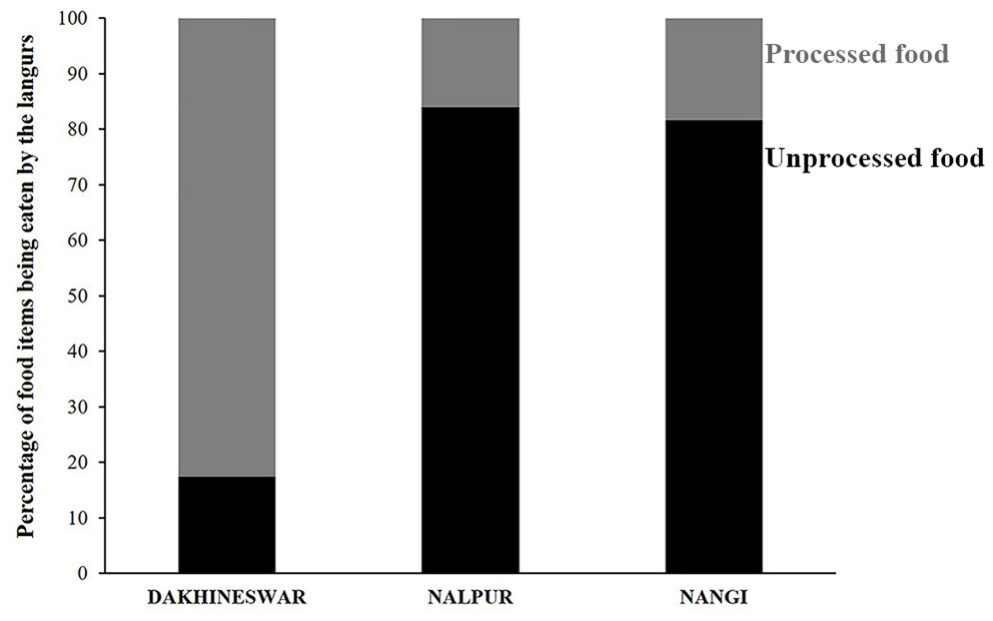
Stacked bar diagram showing the feeding habit of free-ranging langurs at three locations, Dakshineswar, Nalpur, and Nangi. Black and gray bars represent the percentage of “unprocessed” and “processed” food items being eaten by the free-ranging langurs.

We carried out a choice-based experiment between January and March for two consecutive years 2019 and 2020 to find the feeding preferences of free-ranging gray langurs at Dakshineswar where they received human interference, including food offerings (Unpublished data) and depended mostly on “processed foods,” unlike the gray langurs of Nalpur and Nangi where they relied on plant parts such as vegetables, fruits, leaves, flowers (Figure 1). We recorded maximum human provisioning at Dakhineswar between January and March when gray langurs receive both processed and unprocessed food items from the pilgrims (Unpublished data). Therefore, we used this context to explore the feeding preference of free-ranging gray langurs in presence of various human interferences (Bhattacharjee et al., 2020). Here, we conduct the choice-based experiment where we offered a food tray of cardboard, with four types of food items, each of them having a comparable quantity and size (Supplementary Data D4), between 0600 and 1800 h. We used cauliflower and brinjal as “unprocessed food” items, whereas bread and peanuts as “processed food” items. All of these offered food items were fresh and suitable for human consumption. These were presented on the food tray in random order to avoid any “side-bias.” Since peanuts were seen to be one of the most frequently eaten processed food items, we used it for the choice-based experiment and offered it in a small paper bag (which is usually used by people to offer peanuts to gray langurs during provisioning), making its quantity visually similar to the other food items.

### Conducting Experiment at Various Zones of Dakshineswar

Based on the food-census data, we sub-divided the study area, Dakshineswar, into three distinct zones representing various feeding options available to gray langurs (Supplementary Data D5). The experimenter conducted experiments at various zones of Dakshineswar and presented the food tray at a spot where most of the group members could have equal access to the food tray. Since multiple gray langurs visited the food tray during each trial, we considered food items to be our focal object for the analysis instead of gray langurs. The experimenter either waited until the food tray was empty or waited for 10 min if the food tray remained unattended or partially attended by the gray langurs, before closing the session. Once started, the experiment remained undisturbed i.e., no human interference was allowed and the entire experiment was video-recorded. In order to avoid any bias, which could influence subsequent trials, the videos were decoded only in April 2020, after the completion of all experiments. We conducted a total of 83 trials in the field set-up of which 74 trials (where the food trays were attended by the gray langurs without having any human interference) were considered for the final analysis (Supplementary Data D6). Since the movement of free-ranging gray langurs is independent of human supervision, they forage in a group and every group member has easy access to the human-provisioned food items, we did not interfere if more than one gray langur explored the food tray and interact between themselves during food acquisition.

### Scoring Method

For each experiment, we recorded the time points (in seconds) when a food item was attempted to be received by the gray langurs for the first time during a trial i.e., first attempt received by a food item (FA), chosen to be eaten (FC), latency between FA and FC (in seconds) (delay), number of rejection received by a food item (RJ), and the presence or absence of aggression shown by the gray langurs to possess a food item (AG) (Supplementary Data D7). A food item was considered to be rejected if it was not chosen to be eaten followed by FA. One food item could receive multiple RJ between FA and FC (Supplementary Data D7). Then we scored each of the four food items for FA, FC, RJ, AG, and “delay” to check the sequence of food items being picked up and consumed by the free-ranging gray langurs separately for each trial and used these data to reflect the feeding preference of gray langurs (Supplementary Data D7). Since the food tray had four food items, each of them had five scoring options for FA and FC. The food that was attempted to be taken first received a score of five and the last (fourth) one was given a score of two. If a food item remained unattempted, it received a score of one. Similar scoring was done for FC, where the food scored “one” if it was not chosen to be eaten and “five” for being eaten first. RJ was scored on a scale of a maximum of eight to a minimum of “zero,” where food items scored “zero” if they were not rejected at all, and scored “eight” when rejected for FA. Foods were scored “one” if they received aggression and “zero” if not, considering AG as an indication of the possessiveness for the most preferred food item which gray langurs did not want to share with. For “delay” we scored them between “zero” to “five” where “zero” represents no delay, “four” for the maximum delay between FA and FC, and “five” for the foods which were approached but not chosen to be eaten until the end of the experimental session (Supplementary Data D8).

### Statistical Analysis

**Figure 2:**
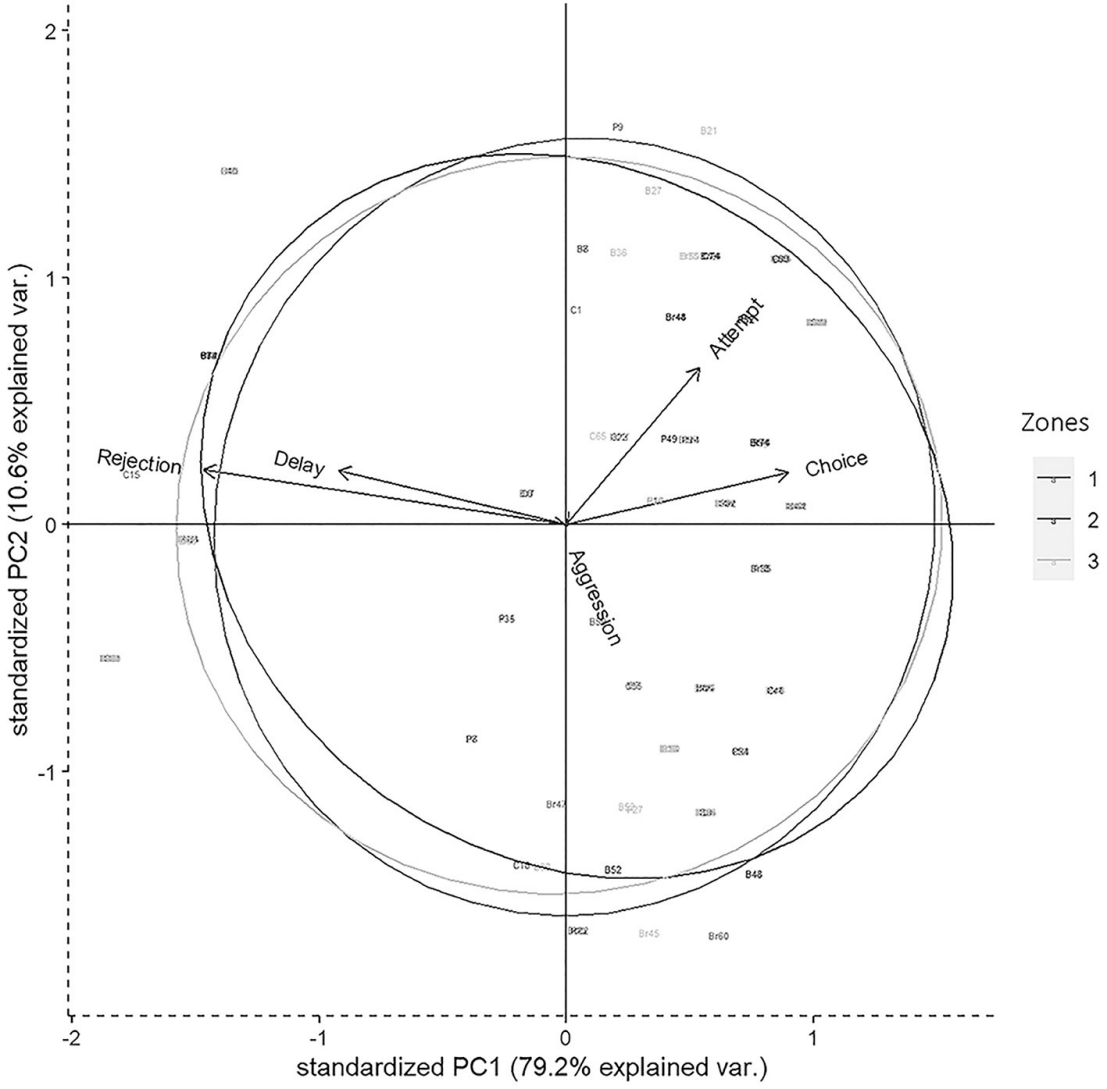
Biplot representing the distribution of variables in 2D space for the Principal component analysis (PCA) having descriptors like attempt, choice, delay, rejection, and aggression. Circles represent three different zones of Dakshineswar.

We used the scores for FA, FC, RJ, AG, and delay for all statistical analyses which were carried out using *StatistiXL* (*version 2.0*), and *R* (*version 4.0.2*) (Team, 2020). We ran a correlation analysis to check the inter-relation between various factors including “attempt” (FA), “choice” (FC), “delay,” “rejection” (RJ), and “aggression” (AG) which were affecting the final food selection by the gray langurs. To verify the results of the correlation we used a generalized linear model (GLM) and checked which parameter was finally affecting the final selection of the food items. We used the FC as the response variable, whereas FA, AG, RJ, and “delay” were incorporated into the model as the predictor variables. We used a “Poisson” distribution for the response variable to run the model. The distribution of the residuals was evaluated to check how well the model fits the data (Supplementary Data D9). A “principal component analysis” (PCA) was conducted for descriptors including FA, FC, AG, RJ, and delay to check their effect on the food selection separately for three zones (Figure 2).

### Multinomial Logit Model

To explain the preference of one food over another, i.e., food choice, we ran varied combination of multinomial logit models (MLM) (Croissant, 2020). Since we were interested in checking the predictive values of different independent variables like aggression, rejection, etc. on the outcome of food choice, we ran two different sets of MLMs – separately for the “attempt” and “choice” probabilities (Table 1). These two sets had four sub-models each where we employed a “leave one component out” (LOCO) approach to meet our goal. The LOCO approach leaves one food component out at each sub-model step to check the order of selection of the subsequent food item. Besides, the models also evaluate the importance of the independent variables or descriptors in the outcome (Supplementary Data D10).

**Table 1:** Table representing the estimates, and *p*-values of the multinomial logit models, set 1 and 2, respectively for (a) attempt, and (b) choice probabilities.

	**Coefficients**	**Estimate**	**Std. Error**	***z*-value**	***p*-value**
**(a)**					
Bread is attempted first					
	Attempt: Brinjal	0.075	0.129	0.580	0.562
	Attempt: Cauliflower	−0.080	0.126	−0.631	0.528
	Attempt: Peanuts	−0.293	0.126	−2.322	0.020 ^*^
Brinjal is attempted first					
	Attempt: Bread	−0.075	0.129	−0.580	0.562
	Attempt: Cauliflower	−0.155	0.128	−1.206	0.228
	Attempt: Peanuts	−0.367	0.128	−2.868	0.004 ^**^
Cauliflower is attempted first					
	Attempt: Bread	0.080	0.126	0.631	0.528
	Attempt: Brinjal	0.155	0.128	1.206	0.228
	Attempt: Peanuts	−0.213	0.124	−1.713	0.087.
Peanut is attempted first					
	Attempt: Bread	0.293	0.126	2.322	0.020 ^*^
	Attempt: Brinjal	0.368	0.128	2.868	0.004 ^**^
	Attempt: Cauliflower	0.213	0.124	1.713	0.087.
**(b)**					
Bread is chosen first					
	Choice: Brinjal	−0.045	0.114	−0.399	0.690
	Choice: Cauliflower	−0.309	0.113	−2.747	0.006 ^**^
	Choice: Peanuts	−0.374	0.113	−3.301	0.001 ^***^
Brinjal is chosen first					
	Choice: Bread	0.045	0.114	0.399	0.690
	Choice: Cauliflower	−0.264	0.111	−2.371	0.018 ^*^
	Choice: Peanuts	−0.328	0.112	−2.935	0.003 ^**^
Cauliflower is chosen first					
	Choice: Bread	0.309	0.113	2.747	0.006 ^**^
	Choice: Brinjal	0.264	0.111	2.371	0.018 ^*^
	Choice: Peanuts	−0.065	0.109	−0.596	0.551
Peanuts is chosen first					
	Choice: Bread	0.374	0.113	3.301	0.001 ^***^
	Choice: Brinjal	0.328	0.112	2.935	0.003 ^**^
	Choice: Cauliflower	0.065	0.109	0.596	0.551

*These two sets represent four sub-models each. Here, we employed a “leave one component out” (LOCO) approach which leaves one food component out at each sub-model step to check the order of selection of the subsequent food item. Significance levels:*

“***” *p: 0.01%;*

“**” *p: 0.05%;*

“*” *p: 0.1%; “·”: 1%; “ ”: not significant*.

The first set used the food attempt as the outcome and the second set used the goal function of final food choice. When one food was attempted to be received by gray langurs, the probabilities of attempting for the next food items can be determined subsequently by using set 1 MLMs. We ran four sub-set MLMs to check what would be the next approached food items separately while considering either brinjal, bread, cauliflower, or peanuts as the “first approached” food item (Table 1a). Higher scores of the odds ratio confirm the results of MLM estimates thereafter (Table 2a) and subsequently rank the different food attempt preferences according to the LOCO tactic.

**Table 2:** Table representing the odds ratio separately for (a) attempt, and (b) choice probabilities.

**(a)**	**Attempt probabilities**		**Odds ratio**
	Bread is attempted first	Attempt: Brinjal	1.078
		Attempt: Cauliflower	0.923
		Attempt: Peanuts	0.746
	Brinjal is attempted first	Attempt: Bread	0.928
		Attempt: Cauliflower	0.857
		Attempt: Peanuts	0.692
	Cauliflower is attempted first	Attempt: Bread	1.083
		Attempt: Brinjal	1.167
		Attempt: Peanuts	0.808
	Peanut is attempted first	Attempt: Bread	1.340
		Attempt: Brinjal	1.445
		Attempt: Cauliflower	1.238
**(b)**	**Choice probabilities**		**Odds ratio**
	Bread is chosen first	Choice: Brinjal	0.956
		Choice: Cauliflower	0.734
		Choice: Peanuts	0.688
	Brinjal is chosen first	Choice: Bread	1.047
		Choice: Cauliflower	0.768
		Choice: Peanuts	0.720
	Cauliflower is chosen first	Choice: Bread	1.362
		Choice: Brinjal	1.302
		Choice: Peanuts	0.937
	Peanuts is chosen first	Choice: Bread	1.453
		Choice: Brinjal	1.389
		Choice: Cauliflower	1.067

**Figure 3:**
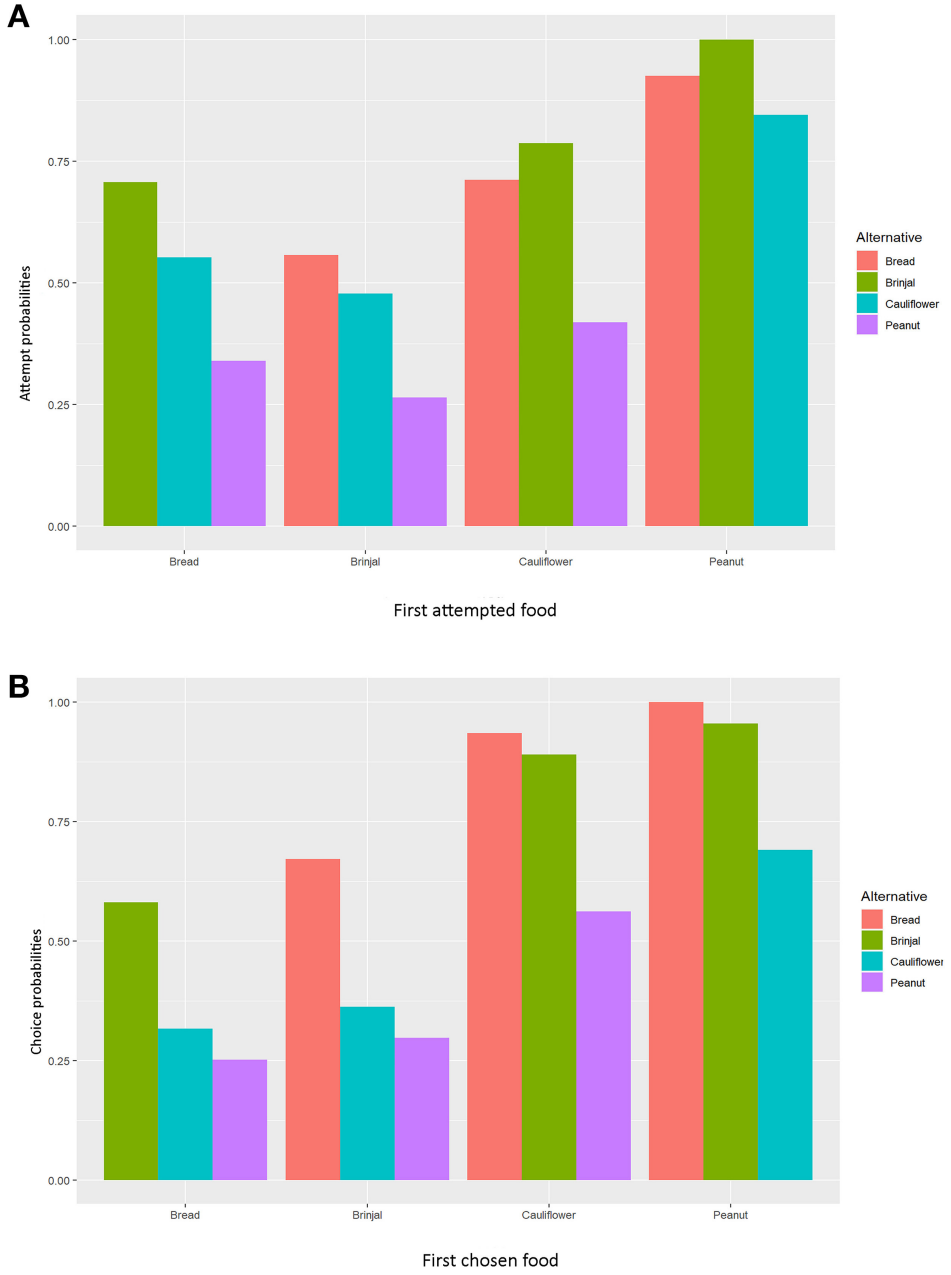
Bar diagrams representing the normalized values of MLM estimates separately for **(A)** “attempt,” and **(B)** “choice” probabilities. The X-axis represents the **(A)** first approached and **(B)** first chosen foods.

Simultaneously, the set 2 MLMs were processed to establish and validate the preferred order of food items being chosen (final food choice) by the gray langurs during the experiment (Tables 1b, 2b). The LOCO here assumes that one food has been consumed (and thus exhausted) and subsequently calculates the probabilities of choosing the next item. Since all food items were provided equally (i.e., equal probability of choice at the beginning), the model considered the frequencies of alternatives equal to 0.25. We used the Newton-Ralphson method from the package “*mlogit*” in *R* to run the MLM (Croissant, 2020). The estimates of the MLM were plotted by using “*tidyverse”* (Wickham et al., 2019) after normalizing to 1.0 (to avoid the negative values) for the visual representation (Figure 3).

### Food Sharing

**Figure 4:**
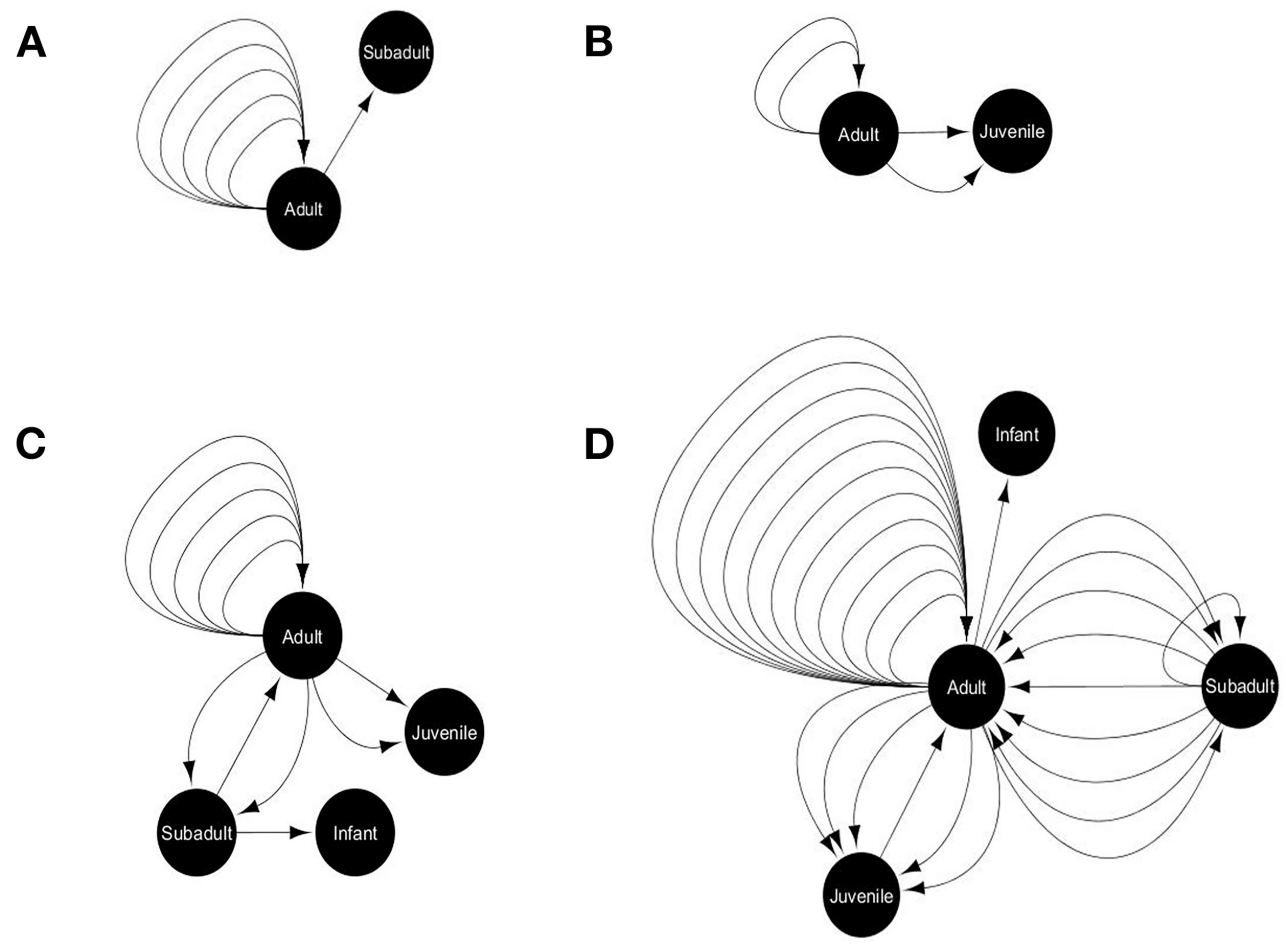
Food-sharing networks of free-ranging hanuman langurs for various food items like **(A)** bread, **(B)** brinjal, **(C)** cauliflower, and **(D)** peanuts. The solid black circle represents a node, depicting a particular life stage of langurs. The black arrow represents one food-sharing behavior between two nodes, which originated from the initiator and directed toward the recipient.

We recorded the incidents of food sharing between gray langurs, if any, out of the total 221 successful cases (where the food items were attended by gray langurs) from a total of 296 cases (four food options for 74 experiments). We used the term “primary recipient” for the gray langurs who received food items directly from the food tray. Food sharing was recorded either when “primary recipient” provided a piece of their food to another group member (secondary recipient), or when the “secondary recipient” attempted to take the food from “primary recipient” and was successful. Therefore, we recorded the details of initiator (who initiated the act), and recipient for each “food sharing” behavior, along with the proportion of food being shared. We used social network analysis (SNA) by using *Cytoscape* (Shannon et al., 2003) where we used various life stages (adult, subadult, juvenile, and infant) as a “node” and an incident of food sharing between them as a “link,” separately for each food type (Figure 4). Here, we calculated the “*indegree*” and “*outdegree*” for each node representing the number of food sharing behavior initiated and received by them respectively.

### Ethical Note

No gray langurs were harmed during this work. All work reported here was purely observation-based and did not involve direct handling of gray langurs in any manner, therefore, was in accordance with approved guidelines of animal rights regulations of the Government of India. The research reported in this paper was sanctioned by DST-INSPIRE, Government of India (approval number: DST/INSPIRE/04/2018/001287, dated 24th July 2018), and was also notified to the Principal Chief Conservator of Forests (PCCF), West Bengal, India.

## Results

### Feeding Habit

Feeding habit of free-ranging gray langurs greatly varied between locations (Contingency chi square: χ^2^ = 122.15, df = 2, *p* > 0.0001). Gray langurs of Dakshineswar largely depended on processed food items (83%) which were mostly human offered (Unpublished data), whereas in Nalpur and Nangi they mostly relied on the plant-based, unprocessed food items (84 and 82%, respectively) (Figure 1).

### Effects of Attempt, Delay, and Rejection to the Final Food Selection

**Figure 5:**
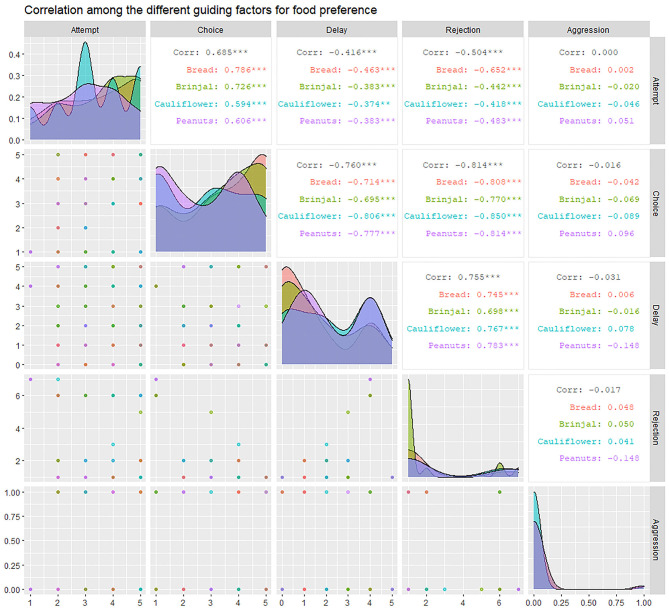
The correlogram representing the inter-relation between factors like “attempt” (FA), “choice” (FC), “rejection” (RJ), “aggression” (AG), and “delay.” It provides the correlation coefficient values (*r*) for each combination of factors and separately for each of the four food items along with their level of significance.

We carried out a total of 83 experimental trials in Dakshineshwar, of which 74 were successful. The experimental outcomes were perused by “Correlation analysis” and “Generalized linear model (GLM).” Correlation analysis- Rejection (RJ) was seen to be highly correlated to attempt (FA), choice (FC), and “delay.” A significant positive correlation (*r* = 0.755, *p* < 0.01) was found between RJ and “delay.” On the other hand, high negative correlations with FA and FC (*r* = FA: −0.504, FC: −0.814; *p* < 0.01) represented inverse relations of the same with these factors. FC was highly and positively correlated to FA (*r* = 0.685, *p* < 0.01), especially toward a few food items like bread and brinjal (bread = 0.786, brinjal = 0.726, cauliflower = 0.594, peanuts = 0.606). On the contrary, “delay” had significant negative effects on FC (*r* = −0.76, *p* < 0.01) (Figure 5).

The GLM confirmed the significant effects of predictor variables like attempt, rejection, and delay on the final food choice. Considering the estimates and *p*-values, while FA (positive) and RJ (negative) showed more significant effects on the FC (*p* < 0.01), “delay” had a lesser impact (negative) (*p* < 0.05) (Table 3). An even distribution of residuals on either side of the “0.0 line” indicated that the model had a good valid fit (Supplementary Data D9).

**Table 3:** Table showing the outcomes of the generalized linear model (GLM).

	**Estimate**	**Std. Error**	***z* value**	***p*-value**
Attempt	0.152	0.031	4.950	7.43e-07 ^***^
Rejection	−0.171	0.028	−6.102	1.05e-09 ^***^
Delay	−0.067	0.030	−2.252	0.0243 ^*^
Aggression	−0.041	0.158	−0.259	0.7957

*“Attempt” shows a positive estimate value for p < 0.01, whereas “rejection” and “delay” come up with negative estimate values for p < 0.01 and p < 0.05, respectively. Though “aggression” has a negative estimate value, it is not significant. Significant codes:*

“***” *p: 0.01%;*

“**” *p: 0.05%;*

“*” *p: 0.1%; “·”: 1%; “ ”: not significant*.

### Influence of Aggression on Food Selection

**Figure 6:**
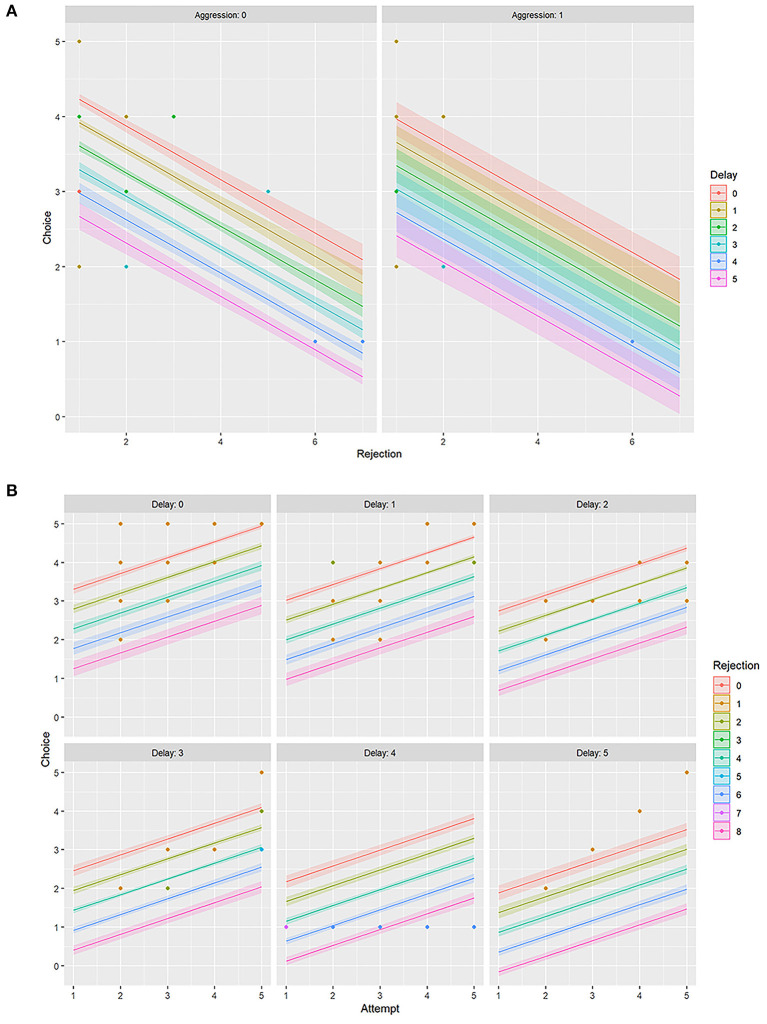
Linear model (LM) plot representing the variations in the choice of food for “rejection” and “delay.” The width of the color bands increases with the delay. **(A)** LM plots showing different levels of “aggression” has different effects on food choice. The left panel represents data for “zero aggression,” whereas the right panel shows that the “presence of aggression” increases the delay in food choice. **(B)** LM plots showing the effects of “attempt” on food choice, together with “delay” and “rejection.” Each panel represents a particular “delay” score. For example, the top left panel is for “no delay” or “zero” delay score, and the bottom right is for the “maximum delay” i.e., score five.

Aggression (AG) had a slight negative influence on both FC and RJ (−0.29 ≤ *r* ≤ 0 i.e., weak negative) (Figure 5, Table 3). However, the linear model (LM) plot revealed that when AG was not present (left panel, aggression = 0) and “delay” was minimum (red color bands), FC was highest for lower RJ and *vice-versa*. Right panel showed that the presence of AG increased the “delay” in FC (width of the color bands represents the “increase”) (Figure 6A). Furthermore, a detailed LM plot revealed that with an increase in the FA, probabilities for FC increased, but both RJ and “delay” lowered the FC (Figure 6B).

### Impact of Descriptors on the Final Food Selection

Results of PCA showed that most of the variability in the experimental observations could be explained through PC1 (79.20%), and subsequently another 10.60% by PC2 (Table 4). The PCA biplot revealed that the “zones” had no impact on food selection by gray langurs. The arrows associated with descriptors “attempt” and “choice” remained close to each other, and pointed in the direction of the increasing values of both PC1 and PC2 (the signs of the eigenvectors are also positive for both PC1 and 2, Table 4, Figure 2), thereby confirming their positive effects on the food selection. However, “delay” almost overlapped with the “rejection” and pointed in the direction of the low value of PC1 but high value of PC2 (the signs of the eigenvectors for PC1 is negative and positive for PC2, Table 4, Figure 2), revealing their negative impact on the food selection. The individual loading of “aggression” was only −0.99 on PC5, therefore considered to have a minimal effect (Table 4).

**Table 4:** Tabulated representation for the principal component analysis (PCA).

	**PC1**	**PC2**	**PC3**	**PC4**	**PC5**
Proportion of Variance	0.792	0.106	0.072	0.026	0.004
Cumulative proportion	0.792	0.898	0.970	0.996	1.000
Loadings of eigenvectors:					
Attempt	0.266	0.859		0.437	
Choice	0.444	0.285	0.153	−0.835	
Delay	−0.456	0.298	−0.789	−0.284	
Rejection	−0.724	0.303	0.595	−0.172	
Aggression					−0.999

*It represents the “proportion of variance” for each principal component (PC), followed by a “cumulative proportion”. It also displays the individual loading for each descriptor like “attempt”, “choice”, “delay”, “rejection”, and “aggression”*.

### Preference for Food Items

The multinomial logit model (MLM) provided a higher score for bread (estimate value: −0.075) among others, revealing the probability of approaching bread as the second alternative, followed by cauliflower (estimate value: −0.155), and peanuts (estimate value: −0.368) when brinjal was attempted first. Similarly, the MLM picked up bread, cauliflower, and peanuts, one by one, as the first attempted food item, and checked the probability of attempt for the rest. Together with MLM estimate values, odds ratio confirmed the highest approach probabilities for brinjal, followed by bread, cauliflower, and peanuts (Tables 1a, 2a, Figure 3A). However, for the choice probabilities, bread scored highest for both the MLM estimates and odds ratio, followed by brinjal, cauliflower, and peanuts (Tables 1b, 2b, Figure 3B).

### First Attempted vs. Eaten Food

Bread and brinjal were chosen as the first attempted food item (scored “five” for FA) for 31 and 32% cases respectively, followed by cauliflower (23%) and peanuts (14%). However, not always the first attempted foods were chosen to be eaten first. Gray langurs switched their preference between the first attempt to first choice for 29.7% cases, and mostly for bread (Goodness of fit: χ^2^ = 31.08, df = 3, *p* < 0.0001) (Supplementary Data D6).

### Food Sharing

Only 18% of the total successful cases were recorded where gray langurs shared the received food items with their troop members during the experimental trials. However, the shared food items mostly consisted of the least preferred peanuts (53%), and cauliflower (22%) (Goodness of fit: χ^2^ = 44.72, df = 3, *p* < 0.0001) (Supplementary Data D11). Social network analysis revealed that food sharing mostly occurs between adults (Goodness of fit: *Outdegree*: χ2 = 75.35, df = 3, *p* < 0.0001; *Indegree*: χ2 = 40.39, df = 3, *p* < 0.0001) and largely for peanuts, and cauliflower (Figure 4).

## Discussion

Folivorous colobines have received considerable research attention because of their unique ability to ingest large quantities of foliage (Struhsaker and Oates, 1975; Oates, 1988; Newton, 1992). Their multipartite stomachs are lined with mucus-secreting glands which facilitate the fermentation of leafy diet in the presence of cellulolytic bacteria (Caton, 1999). However, the dietary composition of free-ranging gray langurs (*Semnopithecus entellus*) seems to be relatively complex. They often use a diverse array of plant parts including leaves, stalks, shoots, buds, flowers, and fruit to utilize the available resources at their best (Yoshiba, 1967; Vandercone et al., 2012). Besides, Srivastava and Winkler added insectivory and human-provisioning to the feeding repertoire of Hanuman langurs (Winkler, 1988; Srivastava, 1989, 1991). However, these feeding habits were mostly seasonal and plant parts still accounted for a significant portion of their regular diet (Koenig and Borries, 2001) similar to the gray langur group of Nungi, and Nalpur.

Surprisingly, the gray langur group of Dakhineswar, West Bengal, India, was spotted to thrive largely (83% of the total diet) on the processed food items for their sustenance within human settlements, throughout the year. Similar to other free-ranging scavengers like dogs, jackals, monkeys (Butler and du Toit, 2002; Sanyal et al., 2010; Paul et al., 2016), this gray langur group was observed to rely upon human generosity and food provisioning, seeking easy access to the anthropogenic food sources (Unpublished data). However, unlike these carnivorous, and omnivorous scavengers, the stomach physiology of gray langur looks alike to that of herbivores such as Macropodidae (Caton, 1999). Therefore, human-provisioned processed foods could have an inevitable health impact, followed by potential behavioral alteration in these urban-adapted free-ranging gray langurs.

Our field-based observational data reflected the highest degree of human interference in Dakhineswar where gray langurs frequently approached humans to acquire processed high-calorie food sources, in contrast to the gray langurs of Nangi, and Nalpur where they opted for foraging and scavenging and depended mostly on plant-based food sources. Therefore, high human-langur interactions could be considered as an intriguing driving force behind altered feeding habits of gray langurs in Dakhineswar. Moreover, the scarcity of plants and crop fields within the urban settlement at Dakhineswar might be another reason behind their consistent dependence on human-provisioned processed food items. In this context, the choice-based field experiment allowed us to understand whether it was the scarcity of plant-based, unprocessed food sources or the easy accessibility of processed food sources that lured them to get accustomed to the urban ecosystem.

The experimental setup allowed gray langurs to choose between processed and unprocessed food items, keeping aside the factors like scantiness, and human influences. Gray langurs chose brinjal, and bread consistently either as the first or second food options, in all the three zones of Dakhineswar, reflecting their feeding preferences within urban settlements. The outcomes of the experiments manifested the “attempt” to be a significant precursor to food selection. Moreover, its close association to the descriptor, “food choice,” for the increasing values of both principal components 1 and 2 in the PCA confirmed the significance of attempt shown by the gray langur toward a particular food item. Therefore, it can be interpreted that the food had to be attempted first prior to the final selection, allowing gray langurs for choice-based decision-making. However, the effects of “rejection” and “delay” were also substantial, and the final food selection by the gray langurs seemed to be non-random but a consequence of all the above three factors. A significant positive correlation between “rejection” and “delay” revealed that more delays in food selection might lead to the ultimate rejection of that particular food item. A greater rate of rejection negatively facilitated the final choice, whereas swifter attempts toward food led to less rejection. Therefore, when an increased “attempt” escalated the probabilities for the final food selection, “delay” gave rise to a dilemma between “food choice” and “rejection” which finally lowered the chances of selection for a given food item. “Aggression” also had some negative effects on both “choice” and “rejection.” Although it increased the “delay” in final food selection, gray langurs used aggression to possess their chosen food items without being forced to share them with other group members. Hence, our experiments revealed that these gray langurs have developed a fondness for both processed and unprocessed food items within an urban settlement at Dakhineswar, which is driven neither by human interference, nor the scantiness of natural food options but by a keen interest in specific food items. The multinomial logit model contemplated all of these factors for the final food selection by the gray langurs and revealed brinjal and bread to be the most attempted food items, followed by cauliflower and peanuts. However, bread outperformed brinjal as the most chosen food item for which gray langurs often switched their first attempted food to the final selection, indicating their inclination to the processed food option.

In the case of food-provisioning where humans provide a food item of their choice to the animals, the animals have no scope to choose but to receive the offered food items. In our experimental setup, gray langurs had the liberty to choose from a platter of offered food items, without any human interference, and the underlying assumption was that the outcome of the experiment would be influenced only by their preference if any. Our findings suggested that even in the presence of plant-based unprocessed vegetables, which is more akin to natural food items of folivorous colobines, these free-ranging gray langurs chose processed food items that were offered in the food-tray. Moreover, the social network analysis revealed that these gray langurs rarely shared bread and brinjal with groupmates, thereby reflecting their comparable fondness for both processed and unprocessed food items. Although the impact of such processed food items on the physiology of gray langurs is still debatable (Maréchal et al., 2016; Geffroy et al., 2017), it can be interpreted that these free-ranging gray langurs of Dakhineswar not only learned to recognize the human-provisioned food items as an alternative to the natural food sources but they displayed behavioral plasticity to take the advantages of anthropogenic habitats which could facilitate their successful survival within an urban ecosystem. However, resource provisioning is often being correlated to the intentions of people to get in touch with the wildlife, imposing a considerable threat to the survival chances of free-ranging animals (Orams, 2002; Trave et al., 2017). Yet, such man-animal interaction opens up possibilities for alternative easy access to resources like food and shelter for these animals who have lost their home due to urban encroachment (Theobald et al., 1997; Lowry et al., 2013; Cox and Gaston, 2018). Moreover, it has been shown that the ability to digest carbohydrates provided ancestral dog populations an advantage over wolves, facilitating the process of domestication, as the dogs could now utilize human-generated resources (Axelsson et al., 2013). Undoubtedly, our experimental results are an example of such urban adaptation where folivorous arboreal gray langurs find their interest in terrestrial processed food items. Therefore, besides their deity value, the survival of free-ranging gray langurs within human settlements and their wide distribution throughout the Indian subcontinent could be well-explained by their altered yet opportunistic feeding pattern.

## Data Availability Statement

Raw data is available at the Dryad data repository. https://doi.org/10.5061/dryad.8cz8w9gpz.

## Ethics Statement

The animal study was reviewed and approved by DST-INSPIRE, Government of India (approval number: DST/INSPIRE/04/2018/001287, dated 24th July 2018).

## Author Contributions

DD, RK, DB, SM, SK, ABh, and SG carried out the field work. MP and DD coded the entire data. ABa and MP carried out all the statistical analyses. ABa prepared the correlation matrix, ran PCA, GLM, and MLM to interpret the data. MP conceptualized the study, got grants to support the work, designed the fieldwork, and supervised the work. MP, DD, and PB drafted the manuscript. PS prepared the GIS map. PB provided the laboratory support to carry out the analysis. All authors contributed to the article and approved the submitted version.

## Conflict of Interest

The authors declare that the research was conducted in the absence of any commercial or financial relationships that could be construed as a potential conflict of interest.
