# Supplementary material for: Altered Food Habits? Understanding the Feeding Preference of Free-Ranging Gray Langurs Within an Urban Settlement
Source: Front Psychol. 2021 Apr 26;12:649027. doi: 10.3389/fpsyg.2021.649027 (PMC8107681; doi:10.3389/fpsyg.2021.649027)
Supplement: Supplementary file 1 [file Data_Sheet_1.docx]

**Supplementary Data**

*Supplementary data SD1*

Map showing the three study locations

*
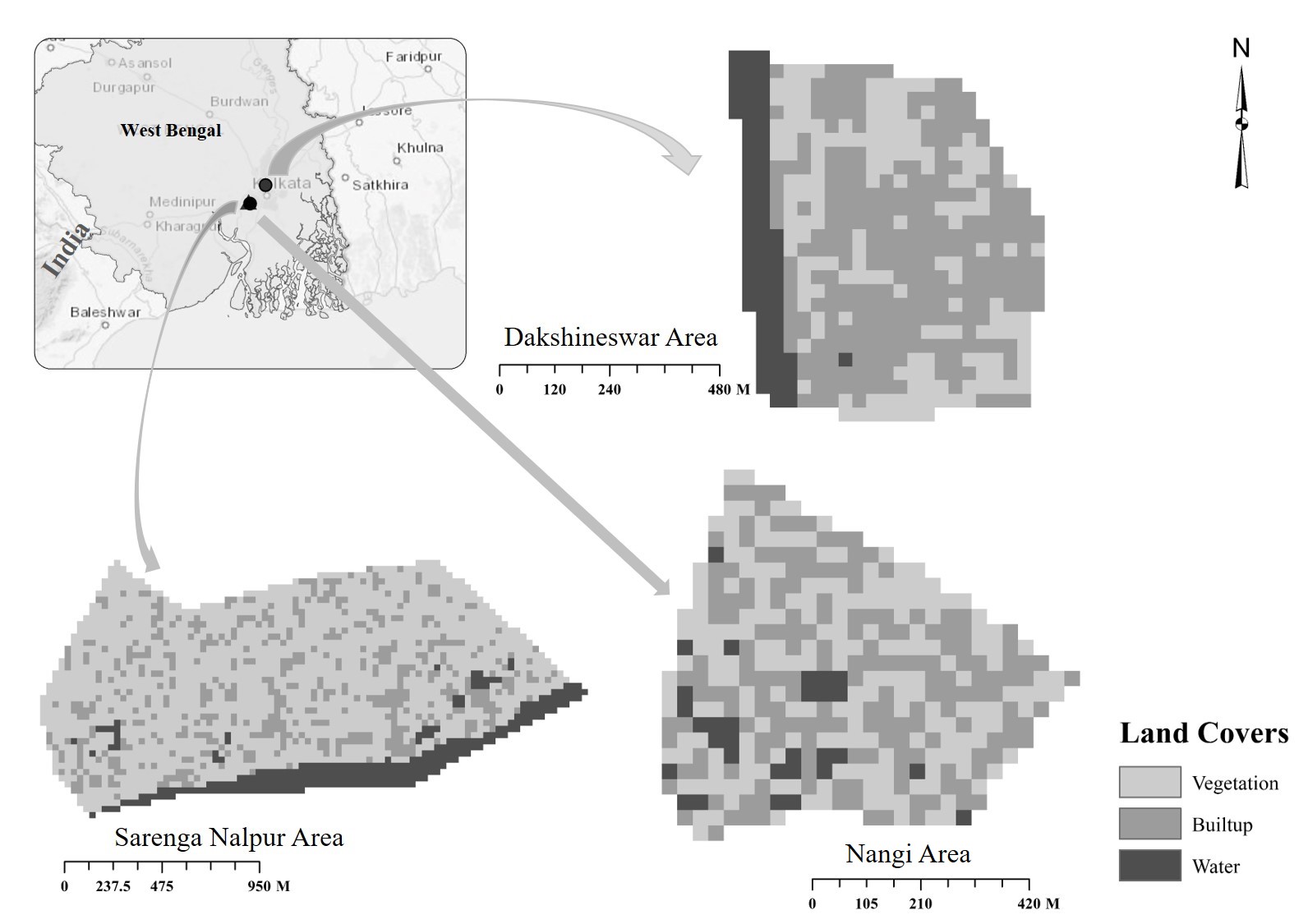
*

*Supplementary data SD2*

Table showing the group composition of three langur troops of Dakshineswar, Nangi, and Nalpur.

| Location | Adult | | Subadult | Juvenile | Infant | Total | Human interferences received  (Average frequency/hr.) |
| --- | --- | --- | --- | --- | --- | --- | --- |
|  | Male | Female |  |  |  |  |  |
| Dakshineswar | 2 | 8 | 2 | 4 | 2 | 18 | Highest (8/hr.) |
| Nangi | 1 | 5 | 3 | 3 | 1 | 13 | Medium (3.8/hr.) |
| Nalpur | 1 | 8 | 5 | 3 | 1 | 18 | Lowest (1.1/hr.) |

*Supplementary data SD3a*

Table showing various types of ‘processed’ and ‘unprocessed’ food items being eaten by the free-ranging langurs of Dakshineswar, Nangi, and Nalpur.

| Sl. No. | Unprocessed food items | Sl. No. | Processed food items |
| --- | --- | --- | --- |
| 1. | Leaves | 1. | Bread |
| 2. | Seeds | 2. | Peanuts |
| 3. | Fruits (both from the tree and market) | 3. | Chips |
| 4. | Vegetables (both from the crop field and market) | 4. | Cake |
| 5. | Tender stems | 5. | Icecream |
| 6. | Roots | 6. | Fried foods |
| 7. | Flowers | 7. | Puffed rice |

*Supplementary data SD3b*

Table shows the calorie values of ‘processed’ and ‘unprocessed’ food items commonly available in the Dakshineswar.

| **Food category** | **Sl. No.** | **Food type** | **Calorie**  **(per 100 grams)** |
| --- | --- | --- | --- |
| Unprocessed | 1. | Leaves | 15 |
|  | 2. | Tomato | 18 |
|  | 3. | Brinjal | 25 |
|  | 4. | Cauliflower | 25 |
|  | 5. | Mango | 60 |
|  | 6. | Guava | 68 |
|  | 7. | Potato | 77 |
|  | 8. | Banana | 89 |
|  |  |  |  |
| Processed | 9. | Bun | 300 |
|  | 10.  11.  12.  13. | Peanuts  Biscuits  Chips  Chocobar | 567 |
|  |  |  |  |

*Site references:*

<http://www.fitbit.com/foods/1+bun/170624>

<http://www.fitbit.com/foods/food?viewFood=on&foodId=698398372>

<https://www.nutritionix.com/food/cauliflower/100-g>

<https://www.nutritionvalue.org/Eggplant%2C_raw_nutritional_value.html>

*Supplementary data SD4*

A ‘pitchboard’ made food-tray having four food items (from top right corner- bread, cauliflower, brinjal, and peanuts) being used for the choice-based field experiment.

*
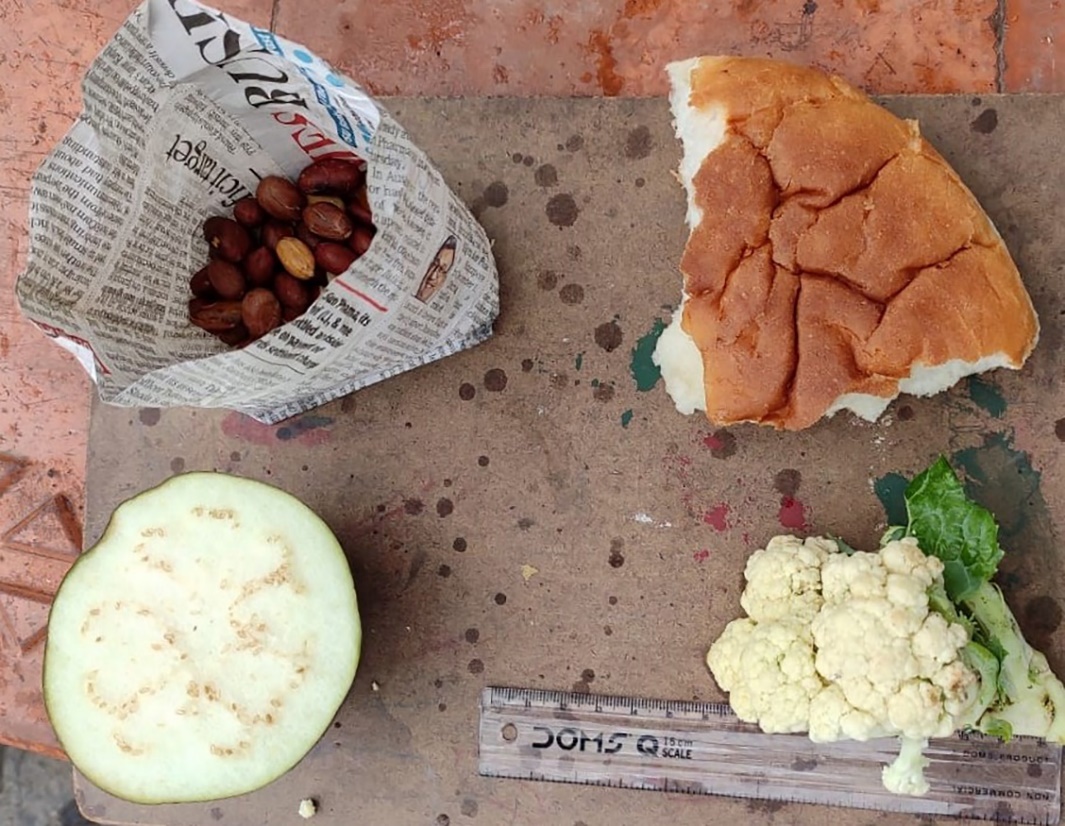
*

*Supplementary data SD5*

Table representing three zones of Dakshineswar. Langurs were observed to depend mostly on human offered food items in ‘zone 1’, whereas in ‘zone 2’ and ‘zone 3’ they depend on both human-offered food items and food items being collected through foraging or scavenging. However, in ‘zone 2’ the foods largely consisted of foraged or scavenged items, but human offered items in ‘zone 3’.

| Zones | Feeding options available to free-ranging langurs |
| --- | --- |
| 1 | Human offerings only |
| 2 | Foraging and scavenging > Human offerings |
| 3 | Human offerings > Foraging and scavenging |

*Supplementary data SD6*

Video of the food choice experiment uploaded separately.

*Supplementary data SD7*

For each experiment, we recorded the time points (in seconds) when a food item was attempted to be received by the langurs (FA), chosen to be eaten (FC), latency between FA and FC (in seconds) (delay), number of rejection received by a food item (RJ), and the presence or absence of aggression shown by the langurs to possess a food item (AG).

Table a. representing detail description of FA, FC, RJ, AG, and ‘delay’.

Table a.

| **Code of the behaviour** | **Name of the behaviour** | **Description** |
| --- | --- | --- |
| FA | A food item attempted to be received | FA represents the time point when a particular food item is being attempted to be received by the free-ranging langurs for the first time during each experimental trial. So, langurs picked up the food item, but not necessarily ate the grabbed item. |
| FC | A food item chosen to be eaten | FC represents the time point when a given food item is being eaten by the free-ranging langurs. |
| Delay | Latency between FA and FC | Delay represents the time duration between FA and FC i.e. the latency between a food item was first attempted to be received and being chosen to be eaten by the langurs. |
| RJ | Rejection | A food item could be first attempted to be recived but not necessarily being selected to be consumed by the langurs. Therefore, rejection refers to the situation when a food item is not chosen to be eaten followed by FA.  A food item could receive multiple rejection between FA and FC. |
| AG | Aggression | During the food acquisition langurs often showed aggressive behavioural responses such as ‘grimace’ (an ugly, twisted facial expression which express disgust), body shaking, exposed teeth, struck, grunting, etc. in order to express their disinterest to allow other group members from food acquisition.  We recorded if a food received any such aggressive behavioural response during its selection by the langurs. |

Table b.

| Food type | FA (sec.) | FC (sec.) | RJ | Delay (sec.) | AG |
| --- | --- | --- | --- | --- | --- |
| Bread | 00.16 | 00.17 | 0 | 1 | 1 |
| Brinjal | 00.13 | 00.29 | 1 | 16 | 0 |
| Cauliflower | 00.21 | 00.35 | 1 | 14 | 0 |
| Peanuts | 00.59 | 01.32 | 3 | 33 | 0 |

Table b. represents the datasheet where we recorded FA, FC, RJ, ‘delay’, and AG for four food items. Although brinjal was attempted first (at the time point 00.13 sec.), bread was chosen first to be consumed (at the time point 00.17 sec.). So here, brinjal received a score of five for FA in contrast to bread which received a score of four. However, for FC, Bread received a score of five and cauliflower four.

We represent the scores in Table c. by using the data from Table b.

Table c.

| Food type | Score | | | | |
| --- | --- | --- | --- | --- | --- |
|  | FA | FC | RJ | Delay | AG |
| Bread | 4 | 5 | 0 | 1 | 1 |
| Brinjal | 5 | 4 | 1 | 3 | 0 |
| Cauliflower | 3 | 3 | 1 | 2 | 0 |
| Peanuts | 2 | 2 | 3 | 4 | 0 |

*Supplementary data SD8*

Table on scoring

| **Behaviour** | **Code** | **Description** | | **Score** |
| --- | --- | --- | --- | --- |
| Attempt received by a food item | FA |  | The food that was attempted first. | 5 |
|  |  |  | The food that was attempted second in the order. | 4 |
|  |  |  | The food that was attempted third in the order. | 3 |
|  |  |  | The food that was attempted fourth in the order. | 2 |
|  |  |  | The food remained unattempted. | 1 |
| Choice i.e a food item chosen to be eaten. | FC |  | The food that was chosen to be eaten first. | 5 |
|  |  |  | The food that was chosen to be eaten second in the order. | 4 |
|  |  |  | The food that was chosen to be eaten third in the order. | 3 |
|  |  |  | The food that was chosen to be eaten fourth in the order. | 2 |
|  |  |  | The food that was not chosen to be eaten during the trial. | 1 |
| Delay or latency between FA and FC for each food item | Delay |  | Food that was attempted to be received but was not chosen to be eaten till the end of a trial. | 5 |
|  |  | If there is any latency (other than ‘zero delay’ situation). | Highest delay | 4 |
|  |  |  | Third lowest delay | 3 |
|  |  |  | Second lowest delay | 2 |
|  |  |  | Lowest delay | 1 |
|  |  |  | No delay between the time points of FA and FC. | 0 |
| Rejection received by a food item between FA and FC. | RJ |  | Food item that was rejected to be attempted i.e received no FA. | 8 |
|  |  | The number of rejections between FA and FC. | Rejected for seven times | 7 |
|  |  |  | Rejected for six times | 6 |
|  |  |  | Rejected for five times | 5 |
|  |  |  | Rejected for four times | 4 |
|  |  |  | Rejected for three times | 3 |
|  |  |  | Rejected for two times | 2 |
|  |  |  | Rejected for one time | 1 |
|  |  |  | No rejection | 0 |
| Aggressive behaviours shown by the langurs to possess a food item | AG |  | Aggression received by the food item. | 1 |
|  |  |  | No aggression received | 0 |

*Supplementary data SD9*

A residual plot for the generalized linear fit model.

*
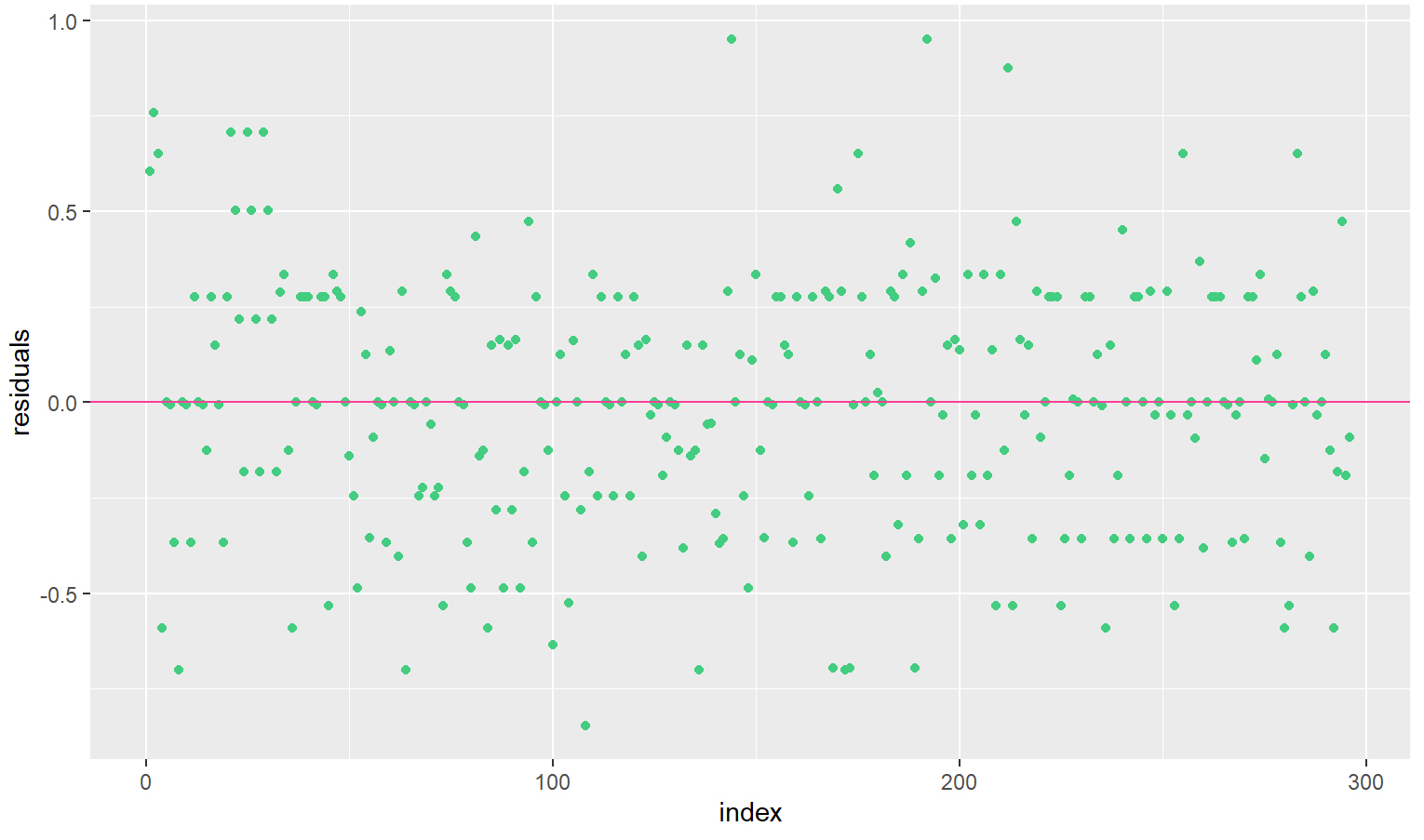
*

*Supplementary data SD10*

| **Model** | **Dependent** | **Independent Descriptor** |
| --- | --- | --- |
| GLM | Choice (FC) | Attempt  Delay  Aggression  Rejection |

Each trial was incorporated into the GLM model as the mixed effect.

| **Model** | **Dependent** | **Alternatives** | **Independent Descriptor** |
| --- | --- | --- | --- |
| MLM | FA ~ Food # | Alternative Food choices | Attempt  Delay  Aggression  Rejection |
|  | FC ~ Food # | Alternative Food choices | Attempt  Delay  Aggression  Rejection |

1. All food items were provided equally to ensure there was no bias regarding available food quantity.

2. Independent variables work together to ensure the final outcome i.e., the marginal effect (which is selected) regarding food attempt or choice.

3. Both dependent and independent variables are categorical – food alternatives.

*Supplementary data SD11*

a) Pie chart representing the percentage of food items being shared between langurs out of the total successful cases. Larger pie on the left side represents the total 296 options of which 221 were successful (black coloured section of the pie). Smaller pie represents the 221 successful cases where the black dotted patch represents cases where the food sharing was observed. b) Pie chart representing the percentage of total food sharing separately for four food items

*
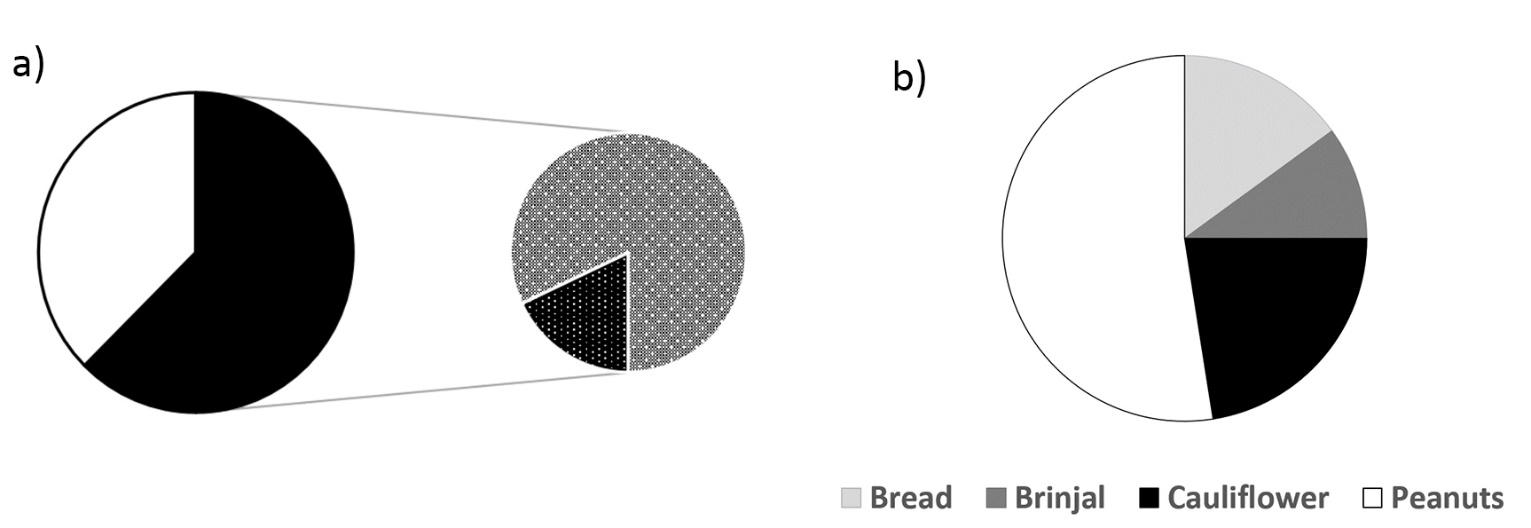
*
